# Physiological audience synchrony in classical concerts linked with listeners’ experiences and attitudes

**DOI:** 10.1038/s41598-024-67455-2

**Published:** 2024-07-16

**Authors:** Wolfgang Tschacher, Steven Greenwood, Christian Weining, Melanie Wald-Fuhrmann, Chandrasekhar Ramakrishnan, Christoph Seibert, Martin Tröndle

**Affiliations:** 1 University of Bern, Bern, Switzerland; 2 Dept. of Cultural Studies, Zeppelin University, Friedrichshafen, Germany; 3 Max-Planck-Institute for Empirical Aesthetics, Frankfurt Am Main, Germany; 4 Illposed, Zürich, Switzerland; 5 University for Music, Karlsruhe, Germany

**Keywords:** Aesthetic experience, Classical concerts, Listening modes, Physiological synchrony, Surrogate synchrony (SUSY), Human behaviour, Cognitive neuroscience

## Abstract

A series of eleven public concerts (staging chamber music by Ludwig van Beethoven, Brett Dean, Johannes Brahms) was organized with the goal to analyze physiological synchronies within the audiences and associations of synchrony with psychological variables. We hypothesized that the music would induce synchronized physiology, which would be linked to participants’ aesthetic experiences, affect, and personality traits. Physiological measures (cardiac, electrodermal, respiration) of 695 participants were recorded during presentations. Before and after concerts, questionnaires provided self-report scales and standardized measures of participants’ affectivity, personality traits, aesthetic experiences and listening modes. Synchrony was computed by a cross-correlational algorithm to obtain, for each participant and physiological variable (heart rate, heart-rate variability, respiration rate, respiration, skin-conductance response), how much each individual participant contributed to overall audience synchrony. In hierarchical models, such synchrony contribution was used as the dependent and the various self-report scales as predictor variables. We found that physiology throughout audiences was significantly synchronized, as expected with the exception of breathing behavior. There were links between synchrony and affectivity. Personality moderated the synchrony levels: Openness was positively associated, Extraversion and Neuroticism negatively. Several factors of experiences and listening modes predicted synchrony. Emotional listening was associated with reduced, whereas both structual and sound-focused listening was associated with increased synchrony. We concluded with an updated, nuanced understanding of synchrony on the timescale of whole concerts, inviting elaboration by synchony studies on shorter timescales of music passages.

## Introduction

Interpersonal synchrony is a ubiquitous phenomenon of social life. People in interaction behave in coordinated ways, and numerous studies of the last years have shown such synchronization on various levels of behavior such as physiological activation^1^, body movement^2^, or prosodic voice qualities^3^. People are typically unaware of their becoming entrained in a conversation, and may even feel awkward when they occasionally find out. Evolutionary biologists have tried to explain this by the possible function of interpersonal synchrony as a kind of courtship dance^4^. In a sociological perspective, synchronous activities may be viewed as a characteristic of social rituals^5^ that strengthen in-group attachment.

Synchrony has also been studied from a different angle, not as a phenomenon of spontaneous entrainment but as resulting from explicit instructions to synchronize and coordinate behavior in experiments. This field of coordination dynamics studied the coordination of movements within^6,7^ and increasingly between participants^8^, finding that stable coordination patterns tended to emerge, which were attributed positively as prosocial in the interpersonal case. This opens up links to the field of music, where joint music-making increases social bonding^9^.

Recent concert studies have now shown that synchrony even occurs in passive listeners of classical music^10,11^. Why should a person’s physiological states vary with the music this person is listening to? As especially in a classical concert we cannot assume that much social interaction is possible between audience members, any measured physiological entrainment among them likely reflects the more or less synchronous individual responses to the music stimulus. The amount of ‘induction synchrony’ was found associated with self-reported aesthetic experiences of immersion in the music, and personality traits of listeners were predictive of the degree of synchrony measured during concerts^12^; however no significant association to affect was found. The goal of the present study was to replicate the significant presence of physiological audience synchrony in a new, larger sample of concert-goers and to study the associations between subjective experiences and the various kinds of synchrony.

Let us briefly recall what physiological signals stand for^13^, since physiological responses to music constitute an active field of research^14^. In general, physiological measures represent bodily activation and capture the activity of the autonomous nervous system (ANS). The ANS has two anatomically and endocrinologically different branches, the activating sympathetic system and the relaxing parasympathetic (also called vagal) system. The peripheral signals that can be measured are the result of sympathetic and/or parasympathetic influence^15^. Two cardiac signals are relatively convenient to record nonintrusively, heart rate (HR, often measured as beats per minute) and heart rate variability (HRV). HR results from both branches of the ANS and higher values denote bodily activation and reduced relaxation. HRV is the subjectively imperceptible variability of the intervals between subsequent heart beats; higher values predominantly reflect parasympathetic activation. Two more signals were recorded in the present study, electrodermal activity and respiratory behavior. Electrodermal activity is the activity of the sweat glands in the skin, which are innervated exclusively by the sympathetic system. The fast component of this activity, skin conductance response (SCR), represents the phasic dynamics of the sympathetic system. Respiration provided two signals for the present research: Respiration rate (RR) is the frequency of breathing cycles, representing both branches of the ANS much like HR; high RR means arousal and activation. The time series of respiratory behavior as such (RESP) indicate at which moment a person inhales and exhales. The mentioned peripheral physiological signals can be conveniently recorded even in a field setting using wearable sensors, and address both the sympathetic and parasympathetic activation of participants. These signals were therefore chosen for the computation of physiological synchrony in the present study.

**Figure 1:**
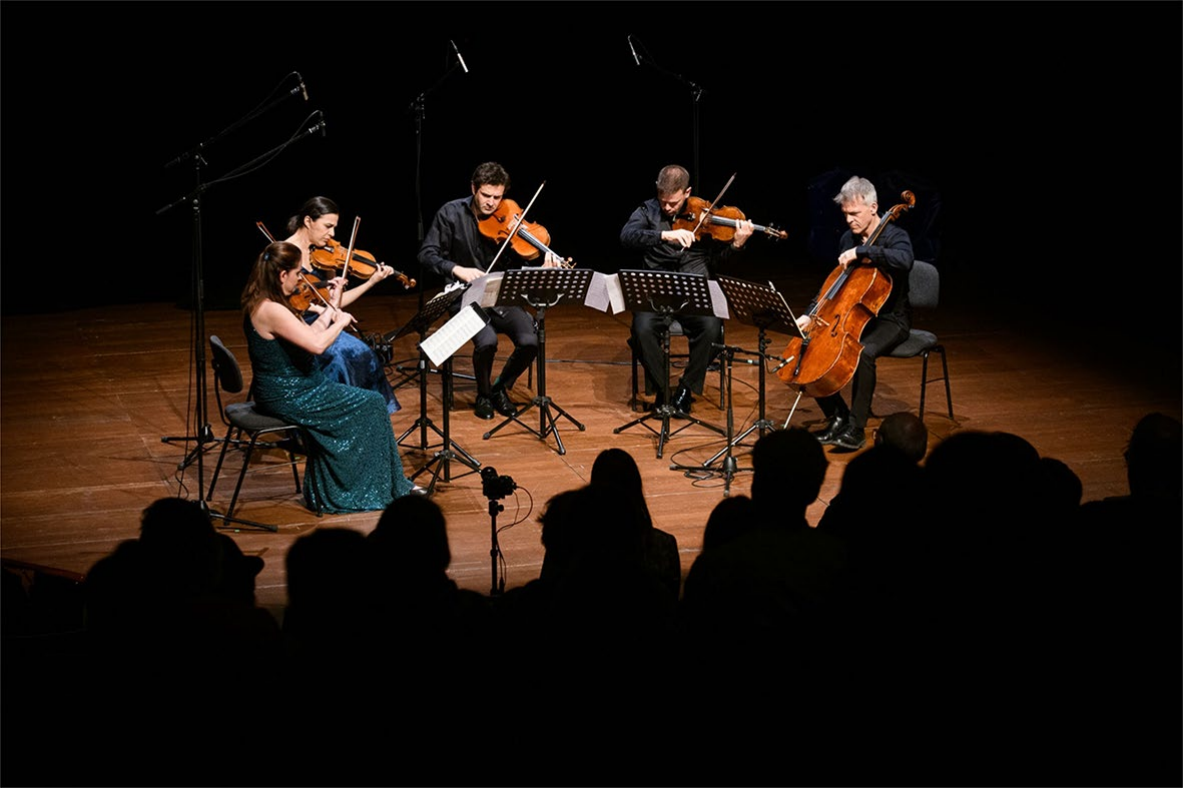
Ensemble “Epitaph” in one of the Radialsystem concerts (Photo: Phil Dera; consent to publish provided by the musicians).

The present study rests on the physiological dynamics recorded in study participants who visited public classical concerts in two renowned concert halls in Berlin, Germany, and the study was organized by the research project “Experimental Concert Research”. A series of eleven concerts was staged in 2022, two concerts in the Pierre Boulez Saal and nine further concerts in the Radialsystem concert hall (Fig. 1). The following pieces were played in all concerts: Ludwig van Beethoven, String Quintet op. 104*;* Brett Dean, *Epitaphs;* Johannes Brahms, String Quintet op. 111. These pieces of Viennese classical, contemporary and romantic Western art music were played partly in different order, and the concert format varied with respect to lighting of the stage, acoustic amplification, or information provided during the concert. The impacts of different formats of staging the music however are not the subject of the present analysis, which focuses in general on the emergence of physiological synchrony during the whole concerts and during the pieces, and further on the associations of synchrony with psychological variables.

The first goal was thus to test the hypothesis H1 that HR, HRV, RR and SCR would be significantly synchronized during the concerts and pieces, whereas, in accordance with previous findings in other samples^12^, the synchronization of phasic respiratory cycles was not expected to arise. Hypothesis H2 concerned participants’ affective states, assuming that synchronies would be associated with affect change during the concerts. It was further expected that synchrony found during the concerts would be linked with listeners’ personality traits^12^, namely with Openness and Agreeableness, and negatively with Neuroticism and Extraversion (H3). Hypothesis H4 focused on the aesthetic experiences addressing the whole concert and the associations of experiences with synchrony (H4). The exact nature of these associations is not known in detail and H4 is thus still exploratory. In analogy to overall concert experience, we also hypothesized that aesthetic experiences specifically concerning the three different pieces would be linked with physiological synchrony retrieved during pieces (H5).

## Results

### Presence of synchrony

Table 1 contains the mean values for all synchrony contributions of all participants in the eleven concerts. Synchrony *contribution* is a measure of how much each single participant contributes to the overall audience synchrony during his or her respective concert and for each piece (for details, see the Methods section). Synchrony contribution is expressed by the aggregated *Z* correlations observed for each participant. These observed *Z* correlations are tested using the distribution of surrogate mean correlations. The *t*-values of Table 1 are then the results of tests of the null hypothesis that observed synchrony *Z* is not different from surrogates *Z*surr. Whenever observed synchrony significantly exceeds surrogates, synchrony is present. All physiological measures HR, HRV, RR, and SCR of participants showed a mean synchronization significantly above surrogates. Synchrony for respiration behavior RESP, which includes the timing of inhalations and exhalations, was not found significantly synchronized, thus no indication was found for synchronized breathing (RESP) beyond synchronized breathing rate (RR). Results thus support hypothesis H1. Owing to the absence of RESP synchrony, we did not consider RESP in later analyses. In a post-hoc analysis of the synchrony differences between the three pieces, we found significant differences of the form Dean > Brahms > Beethoven for HR, HRV and RR synchronies, whereas SCR synchrony in Brahms was significantly higher than both Beethoven and Dean. This post-hoc analysis was based on effect sizes of synchrony not shown in Table 1.

**Table 1:** Physiological audience synchronies across concerts and per piece across concerts.

Variable	Across all concerts	Per piece (across all concerts)		
		Beethoven	Dean	Brahms
HR (*N* = 693)	*Z* = 0.012 (0.008)	*Z* = 0.017 (0.017)	*Z* = 0.012 (0.010)	*Z* = 0.008 (0.008)
	*Z*surr = 0.0002 (0.001)	*Z*surr = 0.0004 (0.003)	*Z*surr = 0.000 (0.001)	*Z*surr = 0.000 (0.000)
	*t* = 39.57****	*t* = 25.81****	*t* = 31.84****	*t* = 27.81****
	ES = 3.27	ES = 1.94	ES = 4.29	ES = 3.59
HRV (*N* = 693)	*Z* = 0.006 (0.009)	*Z* = 0.004 (0.019)	*Z* = 0.010 (0.016)	*Z* = 0.005 (0.011)
	*Z*surr = 0.0005 (0.001)	*Z*surr = 0.001 (0.003)	*Z*surr = 0.000 (0.001)	*Z*surr = 0.000 (0.001)
	*t* = 16.38****	*t* = 4.46****	*t* = 15.77****	*t* = 10.51****
	ES = 4.84	ES = 1.33	ES = 8.32	ES = 5.03
SCR (*N* = 660)	*Z* = 0.133 (0.068)	*Z* = 0.180 (0.114)	*Z* = 0.113 (0.066)	*Z* = 0.107 (0.058)
	*Z*surr = 0.114 (0.063)	*Z*surr = 0.0002 (0.001)	*Z*surr = 0.095 (0.059)	*Z*surr = 0.083 (0.047)
	*t* = 7.48****	*t* = 14.59****	*t* = 7.00****	*t* = 10.85****
	ES = 0.81	ES = 0.75	ES = 0.71	ES = 0.97
RR (*N* = 689)	*Z* = 0.017 (0.017)	*Z* = 0.017 (0.030)	*Z* = 0.020 (0.022)	*Z* = 0.014 (0.017)
	*Z*surr = − 0.0001 (0.001)	*Z*surr = − 0.000 (0.003)	*Z*surr = 0.000 (.002)	*Z*surr = 0.000 (.001)
	*t* = 25.00****	*t* = 15.67****	*t* = 23.81****	*t* = 20.75****
	ES = 4.88	ES = 2.12	ES = 6.99	ES = 5.96
RESP (*N* = 690)	*Z* = 0.000 (0.0003)	*Z* = 0.000 (0.001)	*Z* = 0.000 (0.000)	*Z* = 0.000 (0.000)
	*Z*surr = 0.000 (0.000)	*Z*surr = 0.000 (0.000)	*Z*surr = 0.000 (0.000)	*Z*surr = 0.000 (0.000)
	*t* = − 0.48	*t* = − 0.83	*t* = 1.25	*t* = − 0.00
	ES = 0.00	ES = − 0.00	ES = 0.20	ES = 0.00

*Z* are the means (standard deviations) of all participants’ synchrony contributions. *Z*surr are the mean (standard deviations) of participants’ surrogate synchronies. Mean effect sizes (ES) of synchrony contributions are provided for concerts and pieces. Varying sample sizes *N* due to missings. *t*-tests were conducted to test the distributions of *Z* versus *Z*surr based on the null hypothesis that both are equal.

*HR* heart rate, *HRV* heart rate variability, *SCR* skin conductance response, *RR* respiration rate, *RESP* respiration behavior.

**** p < *0.0001.*

### Changes of affect

The PANAVA items allowed to assess positive affect, negative affect and valence before and after concerts. Table 2 gives an overview of the pre- and post-concert means of affective activation of the various audiences. Pairwise *t*-tests were used to indicate significant changes occurring during the concerts. In eight of eleven concerts, a significant decrease of negative affect was found with an effect size of 0.37 across all concerts. In two concerts positive affect also decreased (across all concerts, the effect size of positive affect was 0.08, which was not significantly different from zero). In the concerts in the Boulez Saal the valence significantly changed towards positive values (across all concerts, the effect size of valence change was 0.10, which is considered a small effect, which was nevertheless significant).

**Table 2:** Descriptive statistics of sample: Means of affective states.

Concert#	PANAVA-PA		PANAVA-NA		PANAVA-VA	
	Pre	Post	Pre	Post	Pre	Post
Boulez 1 (*N* = 68)	0.93	1.03	− 0.97	− 1.62****	1.23	1.71**
Boulez 2 (*N* = 72)	0.94	0.98	− 1.10	− 1.52**	1.38	1.68*
Radial 1 (*N* = 34)	0.66	0.45	− 1.17	− 1.25	1.18	1.22
Radial 2 (*N* = 39)	0.61	0.21*	− 1.10	− 1.64**	1.19	1.32
Radial 3 (*N* = 60)	0.86	1.08	− 1.18	− 1.60**	1.44	1.61
Radial 4 (*N* = 55)	0.57	0.78	− 0.97	− 1.23	1.24	1.44
Radial 5 (*N* = 60)	0.60	0.54	− 1.15	− 1.23	1.48	1.29
Radial 6 (*N* = 74)	0.61	0.50	− 0.79	− 1.39****	1.33	1.42
Radial 7 (*N* = 72)	0.64	0.69	− 0.79	− 1.22**	1.26	1.42
Radial 8 (*N* = 65)	0.53	0.25	− 0.75	− 1.25**	1.30	1.20
Radial 9 (*N* = 84)	0.72	0.38**	− 0.88	− 1.23**	1.25	1.19
All concerts (*N* = 690)	0.71	0.64	− 0.96	− 1.37****	1.30	1.42*

Asterisks indicate concerts with significant pre-post changes (pairwise t-test).

*PA* positive activation, *NA* negative activation, *VA* valence.

*p < 0.05; **p < 0.01; ****p < 0.0001.

Hypothesis H2 postulated that affect changes would be associated with the physiological synchrony measured during the concert. To test this, we fitted hierarchical models to estimate the prediction of synchrony by affect change. In this and the following analyses, physiological synchronies are used as dependent variables, concert number as the random effect that specifies the concert level of our hierarchical dataset, and the predictors (the fixed effects) are the various psychological variables, which in this first analysis was affect change. Affect change was expressed by the effect sizes of positive activation (PA), negative activation (NA) and valence (VA), thus negative effect size of PA indicates increase of positive affect and negative effect size of NA indicates increase of negative affect. Table 3 shows that for two of the four physiological synchronies, namely for HR and SCR, associations were found. HR synchrony was linked to a reduction of NA, whereas SCR synchrony was linked to increase of PA.

**Table 3:** Synchrony contribution and affective change during concerts.

Predictors	HR synchrony		SCR synchrony	
	Estimate	t	Estimate	t
Intercept	3.24	12.4****	0.80	10.1****
PA change			− 0.07	− 2.03*
NA change	0.20	1.99*		
VA change				
Random effect				
Concert (% variance)	9.9		6.1	
*N*	676		644	
r^2^ (% variance)	11.3		8.0	

Results for hierarchical regression of synchrony contribution predicted by PANAVA pre-post change expressed as effect size (i.e., pre minus post/SDpre). Predictors were centered in concert. Only significant predictors are listed, only models with significant predictors are shown. *N* number of observations. r^2^ explained variance of model.

*PA* positive activation, *NA* negative activation, *VA* valence, *HR* heart rate, *SCR* skin conductance response.

*p < 0.05; ****p < 0.0001.

### Synchrony and personality

The short version of the ‘Big Five Inventory’ was filled out before the concert. We again used the five personality traits as predictors of synchrony. Predictors in this modeling approach were not centered as it is implausible that personality traits should have between-concert variance. Hypothesis H3 expressed the expectation that personality would predict synchrony, and this was found for three personality traits and two physiological synchronies and the overall synchrony of all measures (Table 4). HR synchrony was increased in participants with higher Openness (characterizing people who welcome new experiences and have art-related interests) and decreased in extraverted participants, who are rather outgoing and interested in social contacts. HRV synchrony was decreased in people with neuroticistic traits, who tend to be insecure and easily stressed. The link with Extraversion was also found in the synchrony of all synchrony measures taken together (RESP always excluded). The previous finding of Agreeableness predicting synchrony^12^ was however not replicated.

**Table 4:** Synchrony contribution and personality traits.

	HR synchrony		HRV synchrony		All synchronies	
Predictors	Estimate	t	Estimate	t	Estimate	t
Intercept	3.24	12.2****	4.82	12.2****	0.64	9.07****
Extraversion	− 0.37	− 3.58***			− 0.58	− 2.04*
Agreeableness						
Conscientiousness						
Neuroticism			− 0.58	− 2.00*		
Openness	0.38	2.80**				
Random effect						
Concert (% variance)	10.5		3.1		9.3	
*N*	670		670		672	
r^2^ (% variance)	13.9		4.6		11.0	

Results for hierarchical regression of synchrony contribution predicted by personality traits. Only significant predictors listed, only models with significant predictors shown. *N* number of observations. r^2^ explained variance of whole model.

*HR* heart rate, *HRV* heart rate variability.

*p < .05; **p < 0.01; ***p < 0.001; ****p < 0.0001.

### Synchrony and concert experience

The assessment of the aesthetic experience of the concert was based on items of the post-concert survey, which served as predictors of physiological synchronies. To reduce the number of predictors, a factor analysis was performed, and the resulting seven factors were Varimax-rotated to provide orthogonal predictors. All factors were centered in concert. Table 5 shows the associations related to hypothesis H4. The factor F2-emotional listening (give myself over to the music; bathing in the sound; music got under my skin) was associated to synchrony of HR, HRV, RR and the overall synchrony of all physiological signals. This association was always negative in that higher emotional listening was linked with lower synchrony. The factor F5-diffuse distraction (thoughts unrelated to music; listening with half an ear) was likewise associated with lower synchrony of HR, HRV and SCR. Positive associations with synchrony were found for participants high on F3-structural listenening (focusing on the musical structure and on how melodies, rhythm, harmony were composed) and F7-sound (focusing on the sounds, locating sounds in specific instruments).

**Table 5:** Synchrony contribution and concert experience.

Predictors	HR synchrony		HRV synchrony		SCR synchrony			RR synchrony			All synchronies	
	Estimate	t	Estimate	t	Estimate	t		Estimate	t		Estimate	t
Intercept	3.23	12.2***	4.84	11.1***	0.79	10.2***		4.93	9.0***		3.503	12.9***
F1-appreciation												
F2-emotional listening	− 0.29	− 2.77**	− 0.60	− 2.12*				− 0.53	− 2.20*		− 0.38	− 3.62***
F3-structural listening	0.28	2.84**										
F4-atmosphere												
F5-diffuse distraction	− 0.22	− 2.08*	− 0.58	− 2.00*	− 0.16	− 3.75***						
F6-cognitive												
F7-sound			0.69	2.04*							0.33	2.61**
Random effect												
Concert (% variance)	10.5		3.2		6.0		8.6			10.9		
*N*	686		686		654		682			688		
*r*^2^ (% variance)	14.0		6.0		9.3		10.7			14.4		

Results for hierarchical regression of synchrony contribution predicted by experience factors. Only significant predictors are listed. *N* number of observations. *r*^2^ explained variance of random effect and whole model.

*HR* heart rate, *HRV* heart rate variability, *SCR* skin conductance response, *RR* respiration rate.

*p < 0.05; **p < 0.01; *** *p* < 0.001.

### Synchrony and piece experience

The experiences of the three pieces played in the concerts were acquired by nine identical items for each piece after the concert. These items were centered in concert and used as predictors of physiological synchronies in hierarchical modeling. First we modeled the dependent variables of physiological synchronies using all nine predictors, then we repeated the modeling with only the predictors that had become significant to avoid multicollinearity. The items “I liked…”, “I knew…”, “…made me happy”, “…bored me” were not associated with synchrony. Table 6 lists the five predictors significantly associated to synchrony. In the Dean piece, items “was interesting” and “inspired me” were positively predictive of synchrony, the items “annoyed me” and “made me melancholic” predicted decreased synchrony. The item “made me melancholic” had opposite sign in Beethoven and predicted increased synchrony.

**Table 6:** Synchrony contribution and piece experience.

Predictors	Beethoven		Brahms		Dean		Dean		Dean	
	RR synchrony		RR synchrony		HRV synchrony		SCR synchrony		RR synchrony	
	Estimate	t	Estimate	t	Estimate	t	Estimate	t	Estimate	t
Intercept	2.21	6.2***	5.95	0.69	8.08	11.1***	0.69	5.2***	6.98	8.6***
…was interesting					1.63	3.34***				
…inspired me									0.73	2.33*
…moved me			− 0.78	− 2.26*						
…annoyed me							− 0.09	− 2.13*		
…made me melancholic	0.34	2.27*			− 1.35	− 3.04**				
Random effect										
Concert (% variance)	5.9		7.9		2.5		10.3		6.2	
*N*	665		637		644		643		636	
r^2^ (% variance)	6.9		9.6		5.7		11.3		7.3	

Results for hierarchical regression of synchrony contribution predicted by piece-related experience. Only significant predictors are listed. *N* number of observations. r^2^ explained variance of whole model.

*HRV* heart rate variability, *SCR* skin conductance response, *RR* respiration rate.

*p < 0.05; **p < 0.01; ***p < 0.001.

To provide more background to the piece-wise results, we conducted post-hoc analyses of the reported evaluations of the pieces. Hierarchical models were computed to assess the significant differences between pieces finding these in seven of the nine items (Table 7).

**Table 7:** Evaluation and experiences related to pieces (steps of the categorial Piece predictor: Beethoven, Brahms, Dean).

	Liked	Knew	Annoyed me	Moved me	Interesting	Inspired me	Melancholic	Happy	Bored me
*Predictor* Piece (*F* values)	114.2^*s*^	202.9^*s*^	55.2^*s*^	21.5^*s*^	0.87	3.85*	1.70	196.8^*s*^	8.7***
Piece ranks	**A**	**A**	**B**	**C**		**A**		**A**	**B**
Random effect									
Concert (% variance)	1.5	0.9	2.8	1.6	1.2	2.0	2.3	1.7	3.3
*N*	2017	2007	2036	2028	2027	2020	2014	2019	2049
*r*^2^ (% variance)	11.5	17.6	7.9	3.9	1.6	2.6	2.8	17.8	4.2

Results for hierarchical regression of evaluation items (dependent variables) predicted by Piece. *N* number of observations. *r*^2^ explained variance of whole model.Piece ranks (significant differences between pieces): **A**, Beethoven and Brahms > Dean; **B**, Beethoven and Brahms < Dean; **C**, Brahms > Beethoven > Dean.

*p < 0.05; ***p < 0.001; ^*s*^*p* < 0.0001.

## Discussion

The context of the large-scale research project “ECR–Experimental Concert Research” allowed studying physiological synchronies of audiences in a sample that likely constitutes the largest sample in concert studies to date. Almost 700 participants consented to provide physiological recordings and give self-reports on a range of psychological variables and on their experiences related to the presented classical music. Importantly, the participants belonged to the population of common concert-goers, and the eleven included concerts were open to the public. Thus the data were collected “in the wild”, the dataset has considerable ecological validity. One of the main findings of the present study, expressed in hypothesis H1, is that physiological synchrony arises in classical concerts, which is consistent with, and an amplification of, previous findings in independent samples^10,12^. Owing to the conventions of classical concerts—no social interaction in the auditorium during the presentation, subdued lighting in the auditorium—such synchrony within audiences must derive from the shared stimulus, the music. For this reason, the degree of synchrony and the contribution of each audience member to the overall synchrony of the audiences promises to constitute an objective signature of individual responses caused by the music and, in second line, of experiences concerning the music. Synchrony represents the ongoing temporal coordination of listeners’ physiological signals with the music and can thus add further information to the established, commonly cross-sectional, research on the links between music, physiology and listeners’ experiences^14^, which have rarely addressed the dynamical nature of the art form music explicitly. This dynamical nature however is captured in the embodied synchrony of listeners’ physiological dynamics.

We put forward a number of hypotheses to elaborate on the relationships between physiological synchrony and psychological variables. The first expectation (H2) was that synchrony would be linked with affect changes during the concerts. Affect was self-rated before and after concerts. H2 was supported by the finding of higher heart-rate synchrony associated with a decrease of negative activation, and higher electrodermal synchrony with an increase of positive activation. This is a novel finding, as previous hypothesis tests of affect-synchrony associations in a different sample of the ECR project had been insignificant^12^.

The hypothesis of personality traits moderating the individual contributions towards audience synchonies (H3) was likewise supported in the recent dataset. We found Extraversion and Neuroticism negatively linked with synchrony, whereas Openness was positively linked, in replication of the previous research. This shows that personality has an impact on listeners’ attitudes towards music and on how much they ‘go with’ the music. Persons who are socially outgoing and extraverted, or fearful and insecure are less “in-sync” with the audience collective, and persons with higher openness to new experiences and higher aesthetic sensitivity become more synchronized. As Introversion is the opposite pole of the Extraversion scale, one may also state that introverted people become more synchronized.

Hypotheses H4 and H5 concerned aesthetic experiences in relation to the concerts and the different pieces. Concert experience was significantly associated with a number of factors of aesthetic experiencing: Listeners who closely followed the sounds of instruments and who focused on the composition of the music, on melodies, rhythm, and harmony, contributed more to the physiological synchronies. Listeners reporting that they were distracted and listened only “with half an ear” were less synchronized. A further clear finding based on various physiological signals, however, was unexpected: Participants with higher self-reported emotional listening contributed significantly less to audience synchrony, which may appear inconsistent with previous studies^12^ reporting positive associations between emotional immersion and heart-rate synchrony.

Experiences of the single pieces were also linked with the physiological synchrony measured during the pieces. These links were different in each piece, which is plausible since also the evaluations of the pieces and the intercorrelations among the items were quite different. Interestingly, in Brahms the item “moved me” was negatively linked with respiration-rate synchrony, in analogy to the negative link of emotional listening in concert experience.

Our interpretation of findings and associations leans towards a more nuanced understanding of physiological synchrony. In our present data, physiological synchrony was not related to valence and appreciation in general. This follows from the concert experience findings (Table 5) which show no links between synchronies and the appreciation factor and the absence of links with piece-related liking and happiness (Table 6). The links with ‘F3-structural listening’ and ‘F7-sound’ and the negative link with ‘F5-diffuse distraction’ in concert experience also support a clarification of synchrony as the physiological alignment of listeners with the music, in the embodied sense that they continuously and attentively follow the music, irrespective of a positive or negative evaluation of this same music. For example, the Dean piece was generally appreciated the least (Table 7), yet it synchronized the audiences most. The findings concerning personality point in the same direction: People with ‘Openness’ are continuously interested in new experiences, however people with ‘Neuroticism’ tend to ward off new experiences, and extraverted persons are commonly motivated by social (likely rather than musical) experiences. We believe that the initially unexpected findings regarding emotional listening and being-moved suit this interpretation of synchrony: Loadings of items like “give myself over, hide in music”, “listen with feelings”, “music got under my skin”, “bathing in sound” and “touched emotionally” signal moments of exceptional emotional events^16,17^ that can have a rather disruptive quality in the longer run. Hence listeners immersed in this predominantly emotional way risk falling out of sync with the other listeners, who may not experience the same emotions or are following other than emotional listening modes. This interpretation is informed by research and conceptual discussions of the absorption concept in music listening, which show that absorption^18,19^ is a cognitive-attentional and emotional process at the same time. Thus, absorption or absorbed immersion was not covered by the present factorial structure of aesthetic experiences, and the possible relation between absorption and synchrony awaits further research.

We have largely avoided to interpret the various physiological signals the synchronies were computed on. This is due to the cross-correlational processing when synchronies are computed based on these signals. For example, the raw signals of electrodermal activity and heart-rate variability are commonly negatively correlated because they represent sympathetic versus parasympathetic activation—the synchronies of electrodermal activity and heart-rate variability were however minimally correlated in our database. Thus, whereas specific physiological signals carry specific meanings, their synchronies need not. Yet, physiological synchrony of different origin confirms that there is a bodily response to music, supporting the theory that music listening is embodied, maybe predominantly so, and not exclusively cognitive^20^.

Open questions arise from the present findings, which have relied on long pieces of music and even on the durations of whole concerts. The exploration of musicologically well-defined shorter music segments may provide novel insights into physiological responses and their associations to experience. Synchrony can be defined at a hierarchy of timescales^21^ and findings will vary depending on the timescale. Possibly, even respiration behavior may turn out to be in-sync eventually when short timescales are focused as in studying specific excerpts of music. This suggests future studies of the synchronization and experiencing of short music segments.

A further approach to explore audience synchrony can be to compute the synchronies between each listener’s physiological time series and time series of various audio features of the music, such as sound energy or spectral centroid^22^. First results have shown that this approach may open up a second avenue towards exploring physiological synchrony in individuals and in whole audiences, which can directly elucidate which specific acoustic properties make people resonate most with music.

## Methods

### Participants and setting

The research project “ECR—Experimental Concert Research” investigates concert experience from different angles, joining musicology, cultural management and psychology^23,24^. A series of eleven concerts were organized in April and May of 2022 in the two venues Pierre Boulez Saal and Radialsystem in Berlin, Germany. The following pieces were played in all concerts: Ludwig van Beethoven, String Quintet op. 104 in c-minor (only the first movement); Brett Dean, *Epitaphs* (five movements); Johannes Brahms, String Quintet op. 111 in G-major (four movements). Three concerts were played by the Yubal Ensemble, eight by the Ensemble Epitaph.

The concerts were announced by the customary information channels of the respective venue, by mailing lists and by regional media. All concerts were open to the public, but additionally potential audiences were also informed about the possibility to participate in the ECR study and, when interested, were asked to arrive at the venue before the starting time. Participants were then fully informed in verbal and written form about the study procedures, and informed consent was obtained from all participants. The procedure was approved by the ethics committee of the Max-Planck Society, and all research was performed in accordance with relevant guidelines and regulations and with the Declaration of Helsinki. About 75% of the concert audiences consisted of study participants. The present analyses are based on 695 participants with mean age 44.1y (SD = 17.2y). 57.3% of participants were female, 42.2% male, 0.5% other, 4.6% preferred not to say. About 80% of participants had a university degree.

### Self-report measures

Self-report questionnaires were provided before and after each concert. These surveys were filled out by each participant on iPad tablets; LimeSurvey, an open-source software for conducting online surveys (LimeSurvey GmbH, Hamburg) was used to distribute the scales and questionnaires to the iPads and then store the responses on a server. The pre-concert survey acquired demographic data and contained standardized questionnaires for the assessment of affective states, personality traits and music-related attitudes (the latter not used for the present analysis). Affective states were measured using the PANAVA scale, short version^25^, which consists of ten items forming three subscales: positive activation (PA), negative activation (NA), and valence (VA). PANAVA items are bipolar. For example, the item with poles ‘tired’ and ‘wide awake’ loads on PA. The PANAVA subscales have demonstrated good internal consistency with Cronbach’s α ranging from 0.74 to 0.83. Personality traits were assessed by a short form of the ‘Big-five’ personality test also administered before the concert, the 10-items ‘Big Five Inventory’ BFI-10^26^, which is based on five-point Likert scales. Two items are presented for each of the five traits Extraversion, Agreeableness, Conscientiousness, Neuroticism, and Openness. BFI-10 scales were shown to represent the factorial structure of the original Big Five Inventory well, have substantial convergent and discriminant validity.

After the concerts, participants were given the PANAVA again and a number of items to assess music experience in the post-concert survey. Items were used to assess the experience and appreciation of the whole concert (1) and separately of the three presented pieces by Beethoven, Brahms and Dean (2). The latter piece experience items were based on the Aesthetic Emotions Scale (AESTHEMOS,^27^).

(1) Concert evaluation (“Please rate the following aspects of the concert”) was assessed by altogether 15 scales addressing the musicians, the program, the selection and interpretation of pieces, the acoustics and atmosphere of the concert venue on 5-point Likert scales. Eighteen further items (“To what extent did you have these experiences in the concert?”) focused on emotion (touched emotionally, surprised, beautiful, deep connection to the music), cognitive appraisal (made me think, expand my understanding of the music), social involvement (feel connected to others, part of the audience) and disruptive experiences (disturbed by background noises). A battery of 28 items listed various modes of listening (“While listening to the music in the concert…”) based on the modes of cultural consumption elaborated by Rössel^28^, for example: immersed myself in the sound, captivated by the rhythm, musicians’ skill, tried to understand the formal structure, attention to specific instruments, and addressed bodily and emotional responses such as music got under my skin, felt the music physically, felt like crying, listened with feeling. For the purpose of obtaining predictors for ensuing regression analyses, we factorized this large item pool of 61 items. We conducted maximum-likelihood factor analysis with Varimax rotation to reduce the dimensionality of the items to seven factors, which was suggested by the scree plot. Items with communalities < 0.30 were excluded from the final factor model leaving 43 items. The factors explained 46.7% of the variance of the items. Factors were labeled ‘F1-appreciation’ (evaluation of the concert, the musicians, the interpretation), ‘F2-emotional listening’ (give over to the music; “bathing in the sound”; music “got under my skin”), ‘F3-structural listening’ (tried to understand the structure of the pieces; focused on how melodies, rhythm, harmony were composed), ‘F4-atmosphere’ (experienced the atmosphere of the concert; liked the ambience of the venue), ‘F5-diffuse distraction’ (had thoughts unrelated to music; listened with half ear). F6-cognitive (concert made me think; surprised by new impressions; broadened musical understanding) and F7-sound (tried to assign sounds to instruments; tried to identify origins of sounds; focused on sound of instruments).

(2) Appreciation and experience of the three pieces were separately assessed after each concert by nine AESTEMOS items, respectively. The items were “This piece…” “I liked”; “I knew the piece”; “made me angry”; “moved me”; “was interesting”; “made me melancholic”; “made me happy”; “was boring”. The factorial structure differed largely between the three pieces so that all single items were used as predictors in regression models for each piece.

### Physiological measures

**Figure 2:**
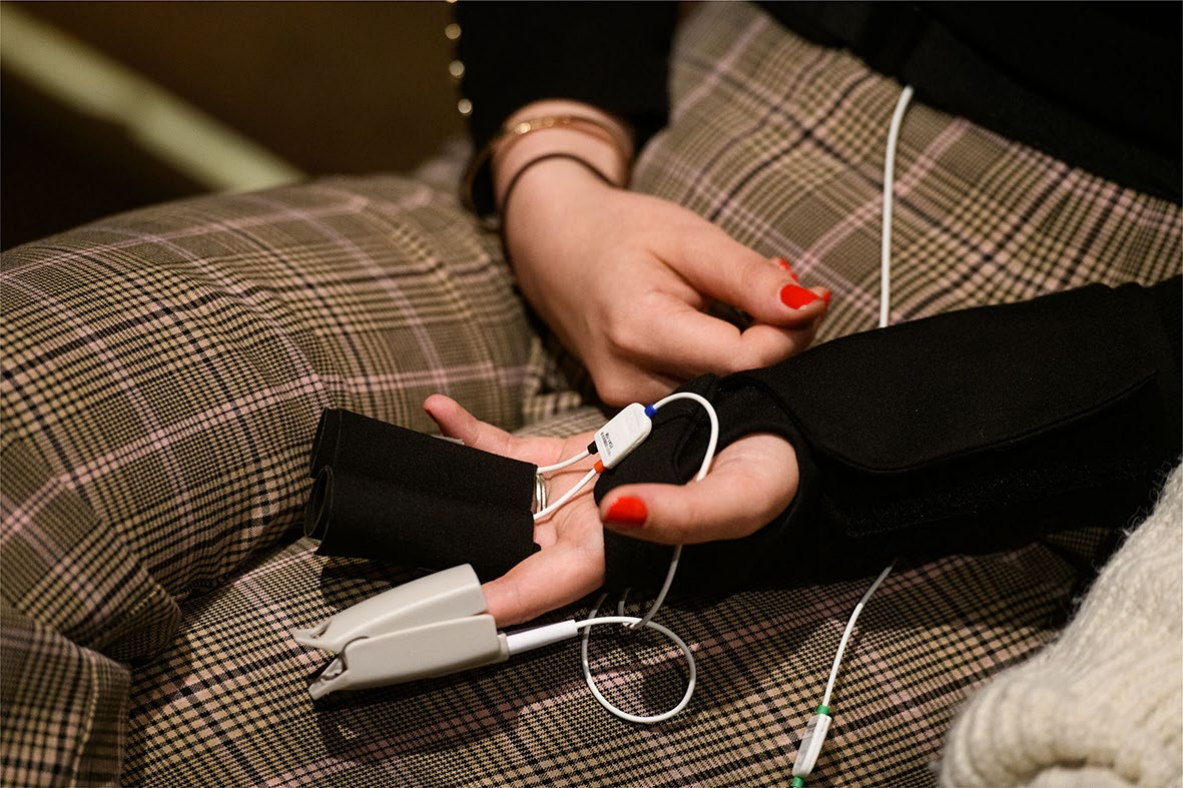
Sensors for physiological recordings (Photo: Phil Dera).

Before the concert, as soon as participants were seated, research assistants equipped each participant with sensors integrated in a glove to collect electrodermal activity, and with a respiration belt to measure breathing (Fig. 2). Devices were manufactured by biosignalsplux (PLUX Wireless Biosignals, S.A.). Physiological data were acquired at 200 Hz sampling rate and were processed using the BioSPPy library^29^ to extract the signals from the raw data of the devices. Blood-volume pulse was captured by a photo-plethysmographic sensor placed over one fingertip. Heart-rate (HR) and heart-rate variability (HRV) were obtained from blood-volume pulse. HRV was computed by the RMSSD (root mean square of successive differences) procedure, which is also applicable to capture short-term variability^14^. Breathing behavior and respiration-rate were derived from belt distension. Electrodermal activity was measured from electrodes attached to two fingers of the non-dominant hand, and pre-processed using Ledalab^30^. In the present analysis, the phasic component of electrodermal activity, skin-conductance response (SCR), was used.

### Synchrony computation

The statistical concept of synchrony means that processes become coordinated and coupled at a level exceeding chance. The raw data are given as time series. We previously developed a correlation-based algorithm, the surrogate synchrony approach (SUSY, available as an R-package:^31^), which assesses the synchrony between two time series based on their cross-correlation function. The standard cross-correlational approach to synchrony is thus dyadic.

Audiences however are multi-person settings and therefore may be described by the ensemble of all dyadic synchronies between the individuals of the audience. Accordingly, audience synchrony is the mean value of all dyadic synchronies within the audience. For example, in an audience of 50 participants, the permutation of all participants is based on 50 × 49 / 2 = 1225 dyadic synchronies. For later use in regression models with individual self-report data, we computed the *contribution* of each single participant A towards the overall audience synchrony, thus in an audience of 50 persons, A’s synchrony contribution is the mean of the 49 dyadic synchronies A has contributed to. Synchrony contribution is thus the individual aspect of the originally interpersonal construct of synchrony.

The statistical synchrony approach of SUSY tolerates and includes lags between people because they may respond differently with regard to musical stimuli (positive lags), or may also anticipate musical events (negative lags). In SUSY the cross-correlations are computed segment-wise; time series were cut into segments of 30 s duration, and the cross-correlations within each segment are computed across a certain range of lags, here we chose maximum lags up to + /– 5 s, so that all cross-correlations in a ten-seconds window, the cross-correlation range *L*, are considered. Segment-size and window-size *L* are basic parameters in SUSY. This operationalization thus includes the simultaneous (*lag* = 0) correlation as well as several time-lagged (cross-) correlations. To allow aggregation of segment-wise cross-correlations, they must be transformed using Fisher’s *Z* transformation. A general measure of synchrony is then mean *Z* across all lags up to + /– 5 s, aggregated inside each segment and then across all segments of the time series. In the present study we used exclusively the non-absolute values of *Z*, which allows to distinguish between in-phase (*Z* > 0) and anti-phase synchrony (*Z* < 0); anti-phase means that one person’s values may be consistently high whenever the other person’s are low. The cross-correlation range of ten seconds means that the psychologically significant ‘moment’ of several seconds, the psychological ‘now’, is covered. This timespan contains between three to seven musical bars, depending on the piece. Thus, as we may assume that phrase-length of the music is commonly four bars, *L* approximately covers a musical phrase.

The second step in SUSY consists of surrogate tests that establish a control condition for mean *Z* by random surrogates. We generated surrogate time series by randomly shuffling the sequence of all segments of a time series. From a dyadic time series with *n* segments, *n*(*n*-1) different surrogate time series can be produced, each of which entails pseudo correlations, as their sequence is falsely arranged by the random mixing of segments. The surrogate step finally generates a signature of synchrony, namely the effect size ES, for non-absolute *Z* cross-correlations, defined as the difference between the ‘real’ *Z* and the mean of all surrogate *Z* (*Z*surr) divided by the standard deviation of the surrogate *Z*. Thus, ES is an effect size at the level of a single bivariate process, which can be aggregated towards the ES of individual synchrony contributions.

### Statistical analyses

Testing the presence of synchrony across concerts and in the pieces within concerts was done using all observed synchrony contributions (expressed by cross-correlations *Z*) and all surrogate *Z* (expressed by the randomly shuffled cross-correlations *Z*surr). *Z* and *Z*surr define two distributions, which were tested against the null hypothesis that they are centered on the same mean value. *Z* exceeding *Z*surr significantly then indicates the presence of synchrony. This test of observed cross-correlations against random surrogate cross-correlations was done separately for each physiological measure (HR, HRV, RR, SCR, RESP) first across the whole concerts and then across each of the three pieces (Beethoven, Dean, Brahms). All statistical procedures were performed using JMP Pro 15.1 software (SAS Institute Inc.).

The various hypotheses on the assumed and hypothesized associations between physiological synchronies and psychological variables (affect, personality, aesthetic concert experiences, aesthetic piece experiences) were examined by hierarchical regression models, with all participants’ synchrony contributions of physiological measures as dependent variables. The predictors (fixed effects) were the respective psychological variables, and ‘concert’ always served as the random effect, because the eleven concerts varied with respect to the concert format. In all models, the random intercept was used. By default we additionally centered the predictors in ‘concert’ in order to disentangle the within-concert variance from the between-concert variance.

## Data Availability

The datasets used and analyzed during the current study are available from the corresponding author on reasonable request.
